# Locus-specific biochemical epigenetics/chromatin biochemistry by insertional chromatin immnoprecipitation (iChIP)

**DOI:** 10.1186/1756-8935-6-S1-P20

**Published:** 2013-03-18

**Authors:** Toshitsugu Fujita, Hodaka Fujii

**Affiliations:** 1 Combined Program on Microbiology and Immunology, Research Institute for Microbial Diseases, Osaka University, Suita, Osaka, 565-0871, Japan

## 

Comprehensive understanding of mechanisms of epigenetic regulation requires identification of molecules bound to genomic regions of interest *in vivo.* However, non-biased methods to identify molecules bound to specific genomic loci *in vivo* are limited. To perform biochemical and molecular biological analysis of specific genomic regions, we developed the insertional chromatin immunoprecipitation (iChIP) technology to purify the genomic regions of interest [1]. The scheme of iChIP is as follows:

(i) A repeat of the recognition sequence of an exogenous DNA-binding protein such as LexA is inserted into the genomic region of interest in the cell to be analyzed.

(ii) The DNA-binding domain (DNA DB) of the exogenous DNA-binding protein is fused with a tag(s) and a nuclear localization signal(s) and expressed into the cell to be analyzed.

(iii) The resultant cell is stimulated, if necessary, and crosslinked with formaldehyde or other crosslinkers.

(iv) The cell is lysed, and the crosslinked DNA is fragmented by sonication or other methods.

(v) The complexes including the exogenous DNA DB are immuno-precipitated with an antibody against the tag.

(vi) The isolated complexes retain molecules interacting with the genomic region of interest. Reverse crosslinking and subsequent purification of DNA, RNA, proteins, or other molecules allows their identification and characterization.

iChIP is a comprehensive approach to purify specific genomic regions of interest to identify interacting molecules including genomic DNA, proteins, RNAs, and others, with an emphasis on non-biased search using next-generation sequencing (NGS), microarrays, mass spectrometry (MS), and other methods. In addition, this approach is not restricted to cultured cell lines but easily extended to organisms *in vivo.*

We applied iChIP to direct identification of components of insulator complexes, which function as boundaries of chromatin domain [2]. We combined iChiP with MS and RT-PCR (iChIP-MS and iChIP-RT-PCR) to find that the chicken beta-globin HS4 (cHS4) insulator complex contains an RNA helicase protein, p68/DDX5; an RNA species, steroid receptor RNA activator 1; and a nuclear matrix protein, Matrin-3, *in vivo*. Binding of p68 and Matrin-3 to the cHS4 insulator core sequence was mediated by CCCTC-binding factor (CTCF). Thus, our results showed that it is feasible to directly identify proteins and RNA bound to a specific genomic region *in vivo* by using iChIP.

In addition, by using the second-generation tagged LexA DB [3], we applied iChIP to identification of proteins interacting with the single-copy *Pax5* locus in the chicken DT40 B cell line. iChIP-MS identified proteins interacting with the *Pax5* promoter using only 4 × 10^7^ cells. Furthermore, iChIP combined with NGS (iChiP-Seq) revealed extensive intra- and inter-chromosomal interactions with the *Pax5* promoter, showing that iChIP-Seq is a powerful technique to detect genome-wide interactions with a specific genomic locus.
